# Genetic analysis of tropical maize inbred lines for resistance to maize lethal necrosis disease

**DOI:** 10.1007/s10681-017-2012-3

**Published:** 2017-09-02

**Authors:** Yoseph Beyene, Manje Gowda, L. M. Suresh, Stephen Mugo, Michael Olsen, Sylvester O. Oikeh, Collins Juma, Amsal Tarekegne, Boddupalli M. Prasanna

**Affiliations:** 1 International Maize and Wheat Improvement Center (CIMMYT), P.O. Box 1041-00621, Nairobi, Kenya; 2 African Agricultural Technology Foundation (AATF), P.O. Box 30709-00100, Nairobi, Kenya; 3 International Maize and Wheat Improvement Center (CIMMYT), 12.5 km Peg Mazowe Road, Mount Pleasant, P.O. Box MP163, Harare, Zimbabwe

**Keywords:** Artificial inoculation, Combining ability, Diallel mating, Maize, MLN resistance

## Abstract

Maize lethal necrosis (MLN) disease is a recent outbreak in eastern Africa and has emerged as a significant threat to maize production in the region. The disease is caused by the co-infection of Maize chlorotic mottle virus and any member of potyviridae family. A total of 28 maize inbred lines with varying levels of tolerance to MLN were crossed in a half-diallel mating design, and the resulting 340 F_1_ crosses and four commercial checks were evaluated under MLN artificial inoculation at Naivasha, Kenya in 2015 and 2016 using an alpha lattice design with two replications. The objectives of the study were to (i) investigate the magnitude of general combining ability variance (σ_GCA_^2^) and specific combining ability variance (σ_SCA_^2^) and their interaction with years; (ii) evaluate the efficiencies of GCA based prediction and hybrid performance by means of a cross-validation procedure; (iii) estimate trait correlations in the hybrids; and (iv) identify the MLN tolerant single cross hybrids to be used as female parents for three-way cross hybrids. Results of the combined analysis of variance revealed that both GCA and SCA effects were significant (P < 0.05) for all traits except for ear rot. For MLN scores at early and late stages, GCA effects were 2.5–3.5 times higher than SCA effects indicating that additive gene action is more important than non-additive gene action. The GCA based prediction efficiency for MLN resistance and grain yield accounted for 67–90% of the variations in the hybrid performance suggesting that GCA-based prediction can be proposed to predict MLN resistance and grain yield prior to field evaluation. Three parents, CKDHL120918, CML550, and CKLTI0227 with significant GCA effects for GY (0.61–1.21; *P* < 0.05) were the most resistant to MLN. Hybrids “CKLTI0227 × CML550”, “CKDHL120918 × CKLTI0138”, and “CKDHL120918 × CKLTI0136” ranked among the best performing hybrids with grain yield of 6.0–6.6 t/ha compared with mean yield of commercial check hybrids (0.6 t/ha). The MLN tolerant inbred lines and single cross hybrids identified in this study could be used to improve MLN tolerance in both public and private sector maize breeding programs in eastern Africa.

## Introduction

Maize is among the most important food crops in the world, and together with rice and wheat, provides over 30% of the food calories to more than 4.5 billion people in 94 developing countries. Maize occupies more than 33 million ha of SSA’s estimated 200 million ha of cultivated land (FAOSTAT 2014). A staple food in many sub-Saharan African countries, maize is grown by millions of resource-poor smallholder farmers. In southern Africa, maize accounts for 77% of the cereal area and 84% of the production, and over 30% of the total calories and protein consumed (FAOSTAT 2014).

Between 2009 and 2011, maize was grown on more than 25 million hectares in sub-Saharan Africa (SSA) (Shiferaw et al. 2011), accounting for 7.5% of global production. Average maize yield in SSA is 1.8 t/ha, which is significantly lower than other maize-growing regions in the developing world. Although several factors including low soil nitrogen, drought, foliar diseases, insect-pests and socio-economic factors contribute to low productivity, recently Maize lethal necrosis (MLN) disease has been one of the major factors affecting maize production in eastern Africa (Mahuku et al. 2015).

MLN disease is caused by the co-infection of two viruses, Maize chlorotic mottle virus (MCMV) and any of the cereal viruses from the Potyviridae family, such as sugarcane mosaic virus (SCMV), maize dwarf mosaic virus (MDMV), or wheat streak mosaic virus (WSMV). MLN was first reported in Kenya in 2011 in the Rift Valley and has subsequently spread to different maize agro ecologies where it is causing considerable losses (Wangai et al. 2012). SCMV was reported several years ago in Kenya (Louie 1980) and South Africa (Handley et al. 1998), but MCMV is new to Africa. MCMV was first identified in Peru in 1973 (Castillo and Hebert 1974) and subsequently reported in the USA, parts of Latin America, and China (Niblett and Claflin 1978; Uyemoto 1983; Xie et al. 2011). MLN has also been reported in Rwanda (Adams et al. 2014) and Democratic Republic of Congo (Lukanda et al. 2014). Similar symptoms on maize have been reported from Uganda and Tanzania (Wangai et al. 2012).

Maize plants are susceptible to MLN disease at all stages of the crop development, from seedling to near maturity. If a maize field is infected early in the cropping cycle, complete yield loss may occur (Uyemoto 1983; Wangai et al. 2012). The USDA Foreign Agricultural Service estimates yield losses in Kenya as high as 10% for the 2014/15 marketing season amounting to over US$50 million (USDA 2014). De Groote et al. (2016) conducted community-survey based assessment on distribution and impact of MLN disease in Kenya and reported 22% of the average maize production in 2013 affected by MLN, with an estimated economic loss of $187 million. The highest yield loss (59%) occurred in the moist transitional zone, with 32% yield loss in the moist mid-altitudes and 15% in the highlands (De Groote et al. 2016).

Insect-pest vectors are the primary mode of plant to plant and field to field transmission. MLN causing viruses can be transmitted by a range of insect vectors endemic in East Africa including thrips (Cabanas et al. 2013) or chrysomelid beetles for MCMV (Nault 1978) and predominantly aphid species for potyviruses (Brault et al. 2010). Both viruses can also be transmitted by seed contamination, mechanism which can contribute to rapid and long-range dissemination of the disease (Jensen 1991; Zhang et al. 2011). Management of MLN in East Africa has been hampered by continuous cultivation of maize throughout the year, lack of resistant maize germplasm and the complicated nature of disease spread and development. Most smallholder farmers in East Africa cannot afford to apply pesticides to control the vector populations, and recycling of seed that may be infected with MLN causing viruses is common in the region.

Development of virus-resistant varieties is an economically viable and environmentally sustainable approach for disease control; however this requires identification of resistant genotypes, and incorporation of the disease resistance into agronomically desirable varieties. In order to screen maize germplasm for resistance to MLN disease, a dedicated MLN screening facility with facilities for artificial inoculation was established jointly by CIMMYT and the Kenya Agriculture and Livestock Research Organization (KALRO) at Naivasha, Kenya, in 2013 (CRP Maize 2013). Since 2013, CIMMYT and its partners have screened more than 95,000 maize germplasm materials including elite inbred lines from CIMMYT and the International Institute of Tropical Agriculture (IITA), maize inbred lines with expired Plant Variety Protection certificates (off-PVP) from USA, experimental three-way and single-cross hybrids, and commercial varieties in eastern Africa under artificial inoculation (AI) at Naivasha. Screening of commercial hybrids in East and Southern Africa (ESA) revealed high levels of susceptibility to MLN (Semagn et al. 2015). As the disease continues to spread, it is crucial that selection for increased tolerance to MLN disease is incorporated into maize breeding programs within SSA.

Maize breeders develop cultivars through cross-breeding of elite inbred lines, and subsequently evaluate them in multiple environments to identify superior cultivars adapted to different agro-ecologies. The mean values of parents and F_1_ combinations are important for estimating combining ability, evaluating performance of hybrids, and selecting superior parents. Identification of best parental combinations is crucial for successful development of MLN resistant hybrids. The diallel design has been widely used for estimating general combining ability (GCA) and specific combining ability (SCA) effects as well as other genetic parameters for grain yield (GY) and other traits (Beyene et al. 2011; Fan et al. 2014). A commonly used diallel analysis procedure that estimates GCA and SCA effects of parents and crosses is Griffing’s method (Griffing 1956). In the current study, 28 inbred parents with varying levels of tolerance to MLN were crossed in a half diallel mating scheme to generate 340 F_1_ hybrids. The 340 F_1_ hybrids along with four widely grown commercial checks were evaluated under MLN artificial inoculation in Naivasha for 2 years. The study includes the first systematic assessment of crosses involving selected MLN tolerant inbred lines in East Africa. The objectives were (i) to investigate the magnitude of general combining ability variance (σ_GCA_^2^), specific combining ability variance (σ_SCA_^2^), and their interaction with years; (ii) to evaluate the efficiencies of GCA based prediction and hybrid performance by means of a cross-validation procedure; (iii) to estimate trait correlations in hybrids; and (iv) to identify MLN tolerant single cross combinations to be used as female parents for three-way cross hybrid production for eastern and southern Africa.

## Materials and methods

### Selection of parents and hybrids formation

Since the establishment of the MLN Screening Facility at the KARLO-Naivasha Research Center in September 2013, a large number of maize germplasm entries have been screened against MLN under artificial inoculation. For this study, a total of 28 inbred parents with varying levels of tolerance to MLN were selected from previous screening results (Beyene et al. unpublished). A total 28 inbred parents (Table 1) were crossed in a half diallel mating scheme and generated 378 F1 hybrids in 2014 at the Maize Research Station of KALRO, Kiboko, Kenya A total of 340 F_1_ hybrids with adequate seeds for evaluation over 2 years were harvested for this study.

**Table 1:** List of the inbred lines used in half-diallel cross and their characteristics

No.	Code	Adaptation	Grain color	MLN reaction
1	CKDHL0159	Drought tolerant	White	Susceptible
2	CKDHL0089	Drought tolerant	White	Susceptible
3	CKDHL120671	Maize streak virus resistant	White	Susceptible
4	CKDHL120694	Maize streak virus resistant	White	Susceptible
5	CKDHL120439	Maize streak virus resistant	White	Susceptible
6	CKDHL120664	Maize streak virus resistant	White	Tolerant
7	CKDHL121310	Insect and maize streak virus resistant	White	Susceptible
8	CKDHL0500	Drought tolerant	White	Moderately tolerant
9	CKDHL120161	Drought tolerant	White	Susceptible
10	CKLMARSI0037	Drought tolerant	White	Tolerant
11	CKLTI0139	Temperate introgression	White	Tolerant
12	CKLTI0227	Temperate introgression	White	Tolerant
13	CKDHL120918	Maize streak virus resistant	White	Tolerant
14	CKLTI0138	Temperate introgression	White	Tolerant
15	CKLTI0137	Temperate introgression	White	Tolerant
16	CKLMARSI0032	Drought tolerant	White	Tolerant
17	CML543	Drought tolerant	White	Moderately tolerant
18	CKLMARSI0022	Drought tolerant	White	Tolerant
19	CKLMLN140479	Drought tolerant	White	Tolerant
20	CML574	Lowland tropical	Yellow	Tolerant
21	CLRCY034	Lowland tropical	Yellow	Tolerant
22	CKLTI0330	Temperate introgression	White	Moderately tolerant
23	CKLTI0136	Temperate introgression	White	Moderately tolerant
24	CML494	Lowland tropical	White	Moderately tolerant
25	CKLTI0318	Temperate introgression	White	Moderately tolerant
26	CKDHL120668	Drought tolerant	White	Moderately tolerant
27	CML550	Low nitrogen tolerant	White	Tolerant
28	CKLMARSI0029	Drought tolerant	White	Moderately tolerant

### Trial design, artificial inoculation and disease scoring

A half-diallel containing 340 experimental F_1_ hybrids plus four commercial checks was evaluated for 2 years under MLN artificial inoculation at Naivasha in 2015 and 2016 using an alpha lattice design with two replications. Entries were planted in two-row plots, 4 m long, with rows spaced at 0.75 m between rows. Two seeds per hill were planted at 0.25 m intervals and stands were thinned to one plant per hill 3 weeks after emergence to obtain a final plant population density of 53,333 plants per hectare. All recommended agronomic management practices were followed.

Maintenance of MCMV and SCMV in susceptible host plants and preparation of MLN inoculum for artificial inoculation was done as reported by Gowda et al. (2015). Maize seedlings were inoculated at 4–6 leaf stage and a second inoculation was conducted 7 days after the first inoculation. A motorized backpack sprayer was used to dispense the inoculum at a rate of 120 L per hectare. 21 and 42 days after inoculation (hereafter known as MLN-early score and MLN-late score, respectively) plants were rated for reaction to MLN using a 1–5 rating scale. In the scale, 1 = clean, no MLN symptom on leaves; 2 = chlorotic mottling on the lower leaves; 3 = chlorotic mottling and mosaic throughout the whole plant; 4 = excessive chlorotic mottling, mosaic, plant necrosis, and/or dead heart; and 5 = dead plant and complete plant necrosis. Other data recorded included grain yield as shelled grains per plot converted to t/ha at 12.5% adjusted moisture content, number of ears per plant (EPP) by dividing the total number of ears harvested per plot by the number of plants at harvest, grain moisture content at harvest and ear rot as number of rotten ears per plot.

### Data analysis

Analysis of variance for each trial and combined analysis across years was performed using the restricted maximum likelihood method of PROC MIXED within SAS 9.2 (SAS Institute 2009). For analysis of total variance, we applied the following linear mixed model:

$$Yijkl{\text{ }}\sim \mu {\text{ }} + {\text{ group }} + {\text{ genotype}}_{i} + {\text{ yr}}_{j} + {\text{ }}\left( {{\text{genotype x yr}}_{{ij}} } \right){\text{ }} + {\text{ rep}}\left( {{\text{yr}}} \right)_{{jk}} + {\text{ blocks}}\left( {{\text{rep x yr}}} \right)_{{jkl}} + {\text{ }}\varepsilon _{{ijkl}} .$$

in which *μ* is the overall mean and group effect includes specific means of genotypes, and checks. The group effect is included in order to avoid inflated estimates of the genotypic variances within the different groups. Genotype_*i*_ is the effect of the *i*th genotype, yr*j* is the effect of the *j*th location, genotype × yr_*ij*_ is the interaction effect of the *i*th genotype by the *j*th year, rep(yr)_*jk*_ is the effect of the *k*th replication within the *j*th year, block(rep × yr)_*jkl*_ is the effect of the *l*th incomplete block within the *k*th replication within the *j*th year, and ε_*ijkl*_ is the residual variance.

The total variance of hybrids was further divided into variance due to general combining ability effects (GCA) of males and females, and variance due to specific combining ability (SCA) of crosses and their interactions with year. Heritability on an entry-mean basis was estimated from the variance components as the ratio of genotypic to phenotypic variance. In addition, best linear unbiased predictions (BLUPs) for each hybrid were calculated across seasons. Phenotypic correlations between MLN and other traits were calculated using combined BLUPs across seasons. For comparing entries evaluated in different years, the entry means were expressed as a percentage of the average performance of the mean of the check hybrids across years.

## Results

### Components of variance–covariance

Analysis of variance showed that genotypic variance and genotype-by-year interaction variance (G × Y) were both significantly (*P* < 0.05) different from zero for all evaluated traits (Table 2). For all traits, except for ear rot and moisture at harvest, the genotypic variance was larger than the genotype-by-year interaction variance, and the highest ratio was for ears per plant followed by GY and MLN severity score at early stage (Table 2). Variation attributed to both GCA and SCA effects was significant (*P* < 0.05) for all traits except for ear rot (Table 2). GCA_Male_ × year interaction was non-significant for all traits except ear rot, while GCA_female_ × year interaction was significant for GY and MLN scores at both stages. Variance of SCA × year interaction were significant for all traits except EPP and ear rot. Estimates of broad-sense heritability ranged from 0.22 for ear rot to 0.73 for late stage MLN severity. MLN severity (resistance) was highly heritable with broad sense heritability of 69–73%. Grain yield had high heritability (0.68), while ear rot and grain moisture had low to intermediate heritability (Table 2).

**Table 2:** Components of variance and heritability for MLN severity scores, grain yield and other agronomic traits of a maize combining ability studies evaluated for 2 years under artificial inoculation

Variance components	MLN-early	MLN-late	GY	EPP	MOI	ER
$\sigma_{\text{G}}^{ 2}$	0.07***	0.10***	0.95***	0.04***	1.56***	8.75*
$\sigma_{{{\text{G}} \times {\text{E}}}}^{2}$	0.03***	0.04***	0.36***	0.01**	2.57***	18.97**
$\sigma_{\text{GCA - Female}}^{2}$	0.03**	0.04**	0.32*	0.02**	0.40*	4.85^ns^
$\sigma_{\text{GCA - Male}}^{2}$	0.04**	0.06**	0.33**	0.02**	0.48**	4.04^ns^
$\sigma_{\text{SCA}}^{2}$	0.01*	0.02**	0.34***	0.01*	0.61*	1.39^ns^
$\sigma_{{{\text{GCA - Female}}*{\text{Year}}}}^{2}$	0.01**	0.02*	0.12*	0.00^ns^	0.22^ns^	5.49^ns^
$\sigma_{{{\text{GCA - Male}}*{\text{Year}}}}^{2}$	0.00^ns^	0.01^ns^	0.07^ns^	0.00^ns^	0.00	8.15*
$\sigma_{{{\text{SCA}}*{\text{Year}}}}^{2}$	0.01*	0.02*	0.21**	0.01^ns^	2.35**	8.16^ns^
$\sigma_{\text{e}}^{ 2}$	0.09***	0.10***	1.01***	0.09***	3.19***	103.45***
GCA:SCA ratio	3.5	2.5	1.0	2.2	0.7	3.2
Heritability (h^2^)	0.69	0.73	0.68	0.62	0.42	0.22

*MLN-early* MLN score at 21 days post inoculation, *MLN-late* MLN score at 42 days post inoculation, *GY* grain yield, MOI percent of moisture at harvest, *EPP* ear per plant, *ER* ears rot

Variance components with their significance levels were given for general combining ability (GCA), specific combining ability (SCA), and interactions with years. The GCA variances were averaged over female and male for calculation of the GCA: SCA variance ratio

*, **, *** *F* test for variance components significant at the 0.05, 0.01 and 0.001 probability levels, respectively

*ns* non-significant at 0.05 probability level

### General and specific combining ability analyses

Four genotypes, CKDHL120918, CKLTI0137, CKLTI0138 and CKDHL0500 had significant negative GCA effects for MLN at early and late disease score intervals (Table 3). CKDHL120918 was most resistant to MLN, with significant GCA effects for GY (1.21, *P* < 0.001), followed by CML550 with positive and significant GCA (0.64, *P* < 0.05), and CKLTI0227 with GCA (0.61, *P* < 0.05). CKDHL120918 had a significant positive GCA effect for ears per plant (0.28, *P* < 0.05) under MLN disease pressure. But CKDHL120694 was the most susceptible to MLN disease with positive and significant GCA effect for MLN severity score at early (0.32, *P* < 0.01) and late stage 0.35 (*P* < 0.01) and negative and significant GCA effects (−0.84, *P* < 0.01) for GY under MLN artificial inoculation (Table 3). The crosses “CKDHL120694 × CKDHL120918”, “CKDHL120918 × CKLTI0136” and “CKLTI0136 × CKLMARSI0029” showed the best SCA effects for grain yield, MLN severity at early and late scores, ears per plant and ear rot (data not shown).

**Table 3:** GCA effect of the 28 inbred lines used in half diallel crosses and evaluated for MLN severity scores, grain yield, and other agronomic traits for 2 years at the Naivasha MLN screening site in Kenya

No.	Pedigree	MLN-early*	MLN-late	GY/t/ha	MOI (%)	EH (cm)	EPP (no)	ER (%)
1	CKDHL0159	0.06	0.07	−0.47	−0.07	−1.97	−0.1*	−0.19
2	CKDHL0089	0.1	0.1	−0.42	1.02*	0.84	−0.11*	−0.61
3	CKDHL120671	0.06	0.1	−0.36	−0.18	−1.28	−0.1*	1.09
4	CKDHL120694	0.32**	0.35***	−0.84**	−0.3	−1.56	−0.34	−0.33
5	CKDHL120439	0.18*	0.21*	−0.69*	0.05	−4.68*	−0.07	0.42
6	CKDHL120664	−0.07	−0.01	0.01	−0.54	−4.52*	0.03	0.65
7	CKDHL121310	−0.13	−0.13	0.1	0.61	4.05	0.01	−0.94
8	CKDHL0500	−0.14*	−0.21*	0.35	0.64	5.88*	0.04	−1.07
9	CKDHL120161	0.15	0.11	−0.62	−0.19	0.85	−0.01	−0.06
10	CKLMARSI0037	0.02	0.03	−0.01	−0.6	−1.47	0.05	−0.44
11	CKLTI0139	−0.09	−0.14	0.09	0.03	0.54	0.1*	−0.58
12	CKLTI0227	−0.13	−0.18*	0.61*	−0.29	2.67	0.14*	−1.59
13	CKDHL120918	−0.47**	−0.54**	1.21***	1.07*	2.25	0.28*	2.4
14	CKLTI0138	−0.19*	−0.19*	0.16	−0.37	−1.07	0.12*	−1.43
15	CKLTI0137	−0.2*	−0.25*	0.29	−0.16	2.49	0.12*	−1.32
16	CKLMARSI0032	0.14	0.16	−0.43	−0.36	−1.7	−0.05	0.66
17	CML543	0.13	0.12	−0.27	0.63	0.35	−0.12*	−0.38
18	CKLMARSI0022	−0.03	−0.01	0.41	−0.26	2.54	0.03	1.92
19	CKLMLN140479	0.17	0.24*	−0.04	−0.35	−4.38	−0.04	0.57
20	CML574	0.05	0.07	0.05	0.09	0.24	−0.08	−0.61
21	CLRCY034	0.02	−0.02	−0.26	0.33	−0.38	−0.14*	−0.62
22	CKLTI0330	0.16	0.17	−0.06	−0.31	1.42	−0.05	0.5
23	CKLTI0136	−0.04	−0.07	0.21	0	−0.41	0.07	−0.61
24	CML494	−0.15*	−0.22	0.5	−0.2	1.51	0.13	−1.64
25	CKLTI0318	0.08	0.15	0.08	−0.21	−0.79	0.02	4.49
26	CKDHL120668	0.09	0.18	−0.24	−0.27	−0.13	−0.02	0.15
27	CML550	−0.11*	−0.1	0.64*	0.21	−1.29	0.08	−0.44
28	CKLMARSI0029	0.01	−0.03	−0.03	−0.76*	−1.73	0.05	0.55

*MLN-early* MLN score 21 days post inoculation, *MLN-late* MLN score 42 days post inoculation, *GY* grain yield, *MOI* percent of moisture at harvest, *EPP* ear per plant, *ER* ears rot

*, **, *** F test for variance components significant at the 0.05, 0.01 and 0.001 probability levels, respectively

### Prediction based on general combining ability and hybrid performance

**Fig. 1:**
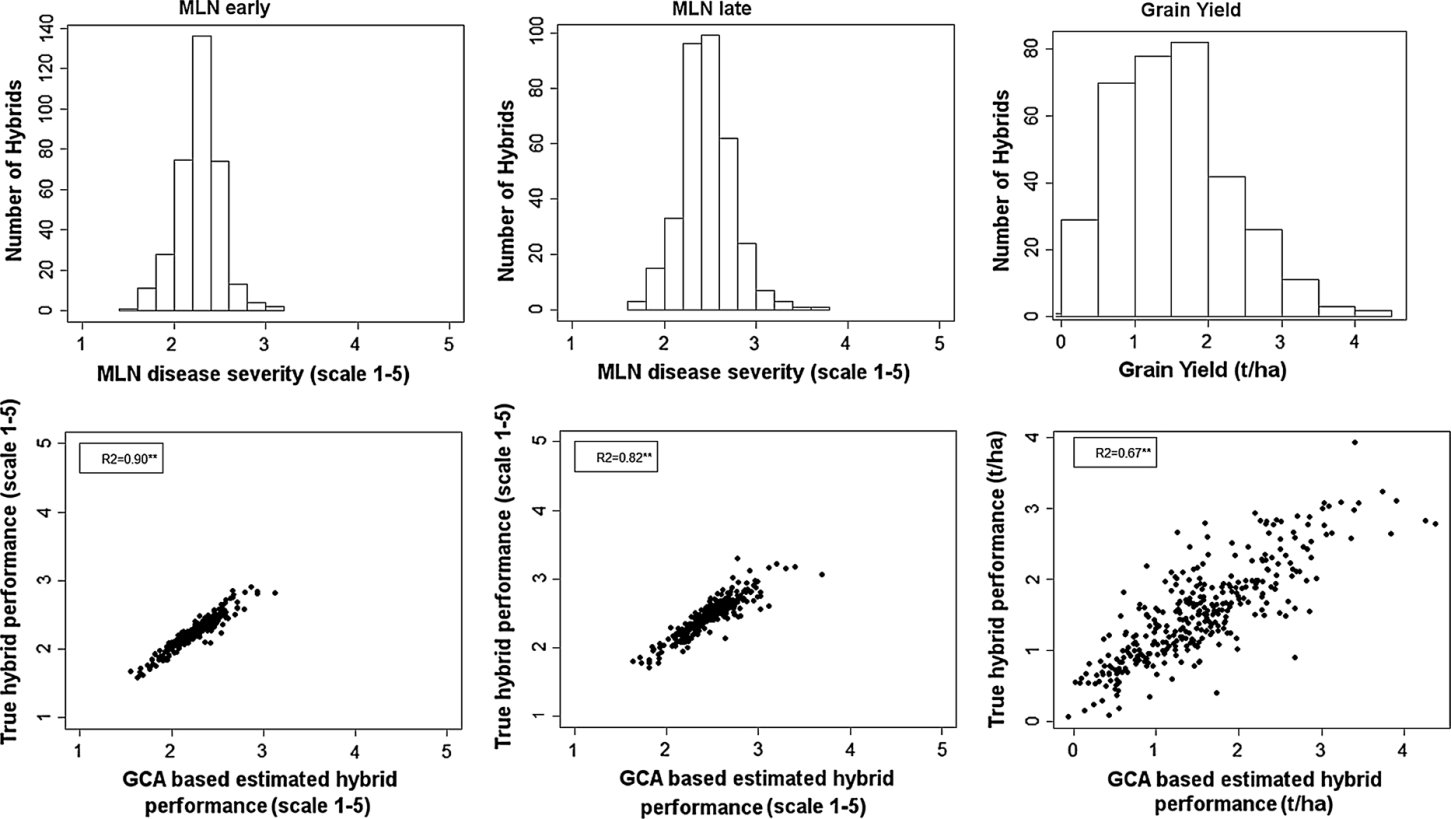
MLN severity and grain yield frequency distribution (*top*) and GCA based prediction for MLN severity at early and late stage and grain yield of 344 hybrids evaluated for 2 years in Kenya

General combining ability based prediction for MLN severity at early and late stages, GY and their levels of significance was shown in Fig. 1. Estimates of GCA prediction efficiency (R^2^) and hybrids performance were highly significant (P < 0.01) for all traits. The GCA based prediction efficiency (R^2^) explained 67–90% of the observed GCA variations for GY, MLN-late and MLN-early. Grain yield, early and late MLN severity were normally distributed (Fig. 1).

### Mean performance of hybrids

**Fig. 2:**
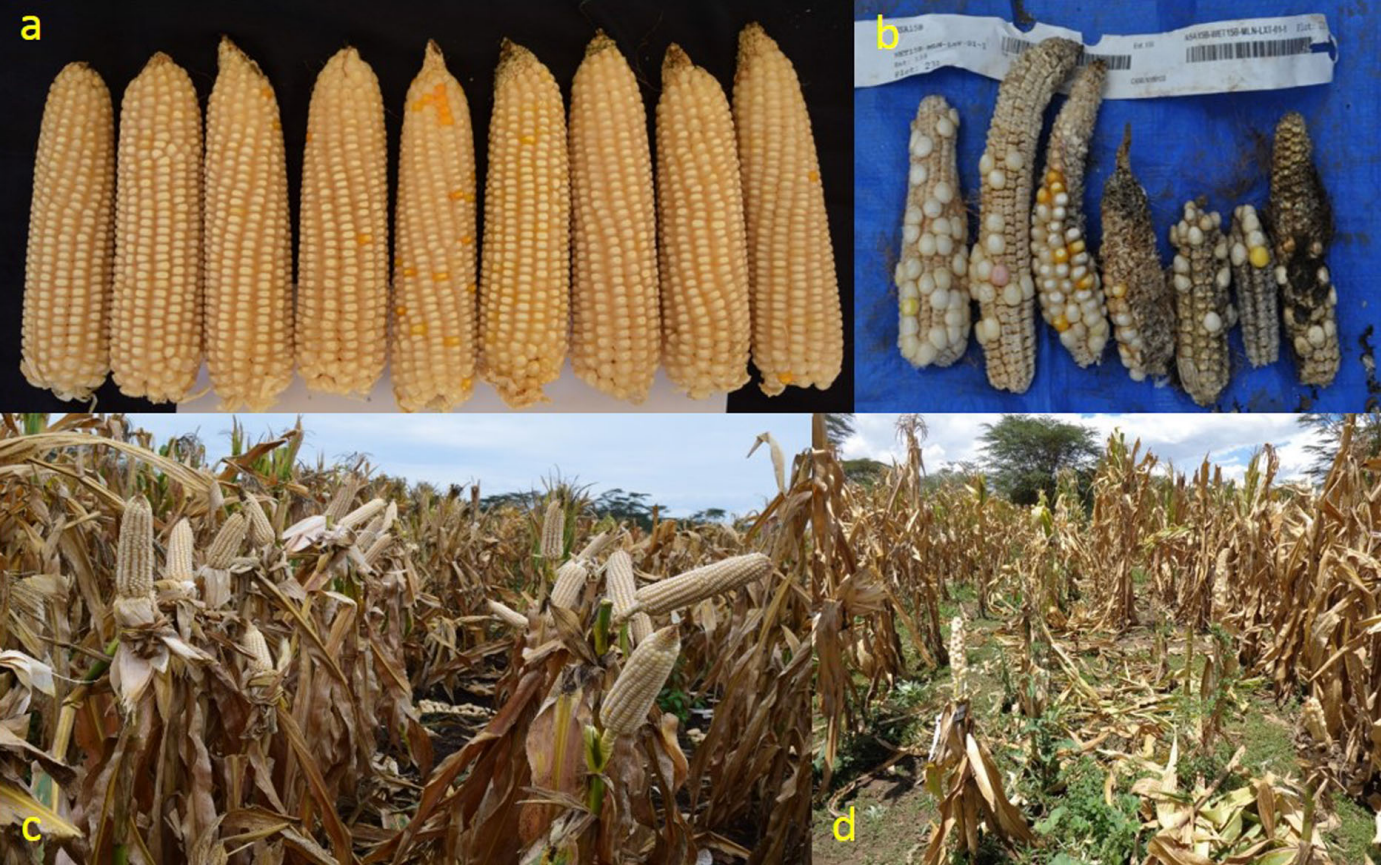
MLN-resistant hybrid versus susceptible commercial check. **a** ears of a MLN-resistant hybrid at harvest; **b** ears of a MLN-susceptible commercial check; **c** a MLN-resistant hybrid (just before harvest) at the MLN screening facility; **d** a susceptible commercial hybrid check (at the same stage)

MLN disease severity, GY, and other agronomic traits for the best 30, worst five single crosses, and four commercial checks are shown in Table 4. Responses of genotypes to MLN under artificial inoculation were striking among the MLN-resistant hybrids and susceptible commercial checks at Naivasha (Fig. 2). Mean GY varied from 0.20 t/ha for DUMA43 (commercial check) to 6.6 t/ha for hybrid “CKLTI0227 × CML550” (experimental hybrid) with an overall trial mean of 2.04 t/ha. Hybrid “CKLTI0227 × CML550” was the best performing hybrid in GY (6.6 t/ha) across years, followed by “CKDHL120918 × CKLTI0138” (6.5 t/ha), “CKDHL120918 × CKLTI0136” (6.0 t/ha), “CKLTI0227 × CKDHL120918” (5.4 t/ha) and “CKLTI0227 × CML550” (5.0 t/ha) while the best commercial check hybrid (WE1101) had GY of 0.9 t/ha. The top 10 hybrids produced 684–1008% higher GY than the mean of the commercial checks (Table 4). The mean MLN scores of the top 10 MLN-tolerant hybrids at early stage (2.1) and late stage (2.3) were lower than the mean of commercial checks at early stage (3.1) and at late stage. (3.4). On average, the commercial checks had the highest (20.6%) ear rot infection while the top 10 MLN tolerant hybrids had the least (2.3%). The top 10 MLN tolerant hybrids had the highest (1.3) number of ears per plant while the commercial checks had the least (0.5).

**Table 4:** Mean grain yield, MLN disease severity, and other agronomic traits of the top 30 hybrids, the lowest five experimental hybrids and commercial checks evaluated under artificial MLN inoculation in 2015 and 2016 at Naivasha, Kenya

Entry number	Hybrids	GY (t/ha)	% increase over the mean of checks	MLN-early (1–5)	MLN-late (1–5)	MOI (%)	EPP (number)	ER (%)
1	12 × 27	6.6	1008.3	2.3	2.5	20.3	1.3	2.0
2	13 × 14	6.5	990.4	2.0	2.3	16.6	1.7	1.0
3	13 × 23	6.0	896.9	1.9	1.9	22.4	1.6	0.7
4	12 × 13	5.4	792.4	1.6	1.8	21.1	1.5	1.0
5	12 × 27	5.0	733.9	2.3	2.4	18.9	1.3	0.1
6	18 × 24	4.9	713.2	2.5	2.6	16.6	1.2	1.2
7	4 × 13	4.8	698.5	2.1	2.1	21.9	1.1	1.0
8	11 × 13	4.8	696.2	1.9	2.1	23.2	1.3	6.2
9	13 × 24	4.8	693.7	2.3	2.3	21.8	1.3	0.3
10	6 × 23	4.7	684.4	2.3	2.4	16.3	1.1	9.4
Mean of the top 10 entries		5.3	790.8	2.1	2.3	19.9	1.3	2.3
11	23 × 28	4.5	643.5	2.3	2.8	18.0	1.3	2.3
12	13 × 28	4.4	633.3	1.9	2.1	19.0	2.2	3.0
13	12 × 17	4.4	632.5	2.5	2.7	19.4	1.1	0.2
14	24 × 27	4.4	626.7	2.5	2.5	18.2	1.3	0.1
15	27 × 28	4.4	625.4	2.4	2.5	19.5	1.3	1.4
16	10 × 27	4.3	624.9	2.3	2.5	17.2	1.3	0.1
17	13 × 18	4.3	619.0	1.7	1.9	16.9	1.4	37.1
18	15 × 17	4.3	618.9	2.7	2.7	17.3	1.0	0.1
19	8 × 13	4.3	612.2	2.1	2.2	23.0	1.2	1.7
20	12 × 18	4.2	608.0	2.2	2.7	18.6	1.2	1.5
21	11 × 27	4.2	599.6	2.5	2.7	17.2	1.0	0.7
22	8 × 14	4.2	595.5	2.2	2.2	21.4	1.1	1.3
23	8 × 18	4.1	587.6	2.3	2.4	21.2	1.2	0.1
24	10 × 11	4.1	584.0	2.2	2.5	16.3	1.0	0.4
25	14 × 19	4.1	583.0	2.3	2.6	15.4	1.3	2.3
26	17 × 27	4.1	578.8	2.6	2.9	20.5	1.1	2.3
27	8 × 12	4.0	569.6	2.1	2.5	20.2	1.1	0.1
28	10 × 12	4.0	566.4	2.0	2.4	18.1	1.2	0.5
29	8 × 24	4.0	563.8	2.2	2.4	22.1	1.2	0.1
30	15 × 25	4.0	563.3	2.1	2.8	17.3	1.2	4.1
31	19 × 25	0.0	−100.0	3.1	3.7	18.8	0.6	0.8
32	1 × 7	0.0	−100.0	3.4	4.1	18.7	0.2	3.6
33	22 × 25	0.0	−100.0	2.7	4.2	18.6	−0.1	4.2
34	2 × 9	0.0	−100.0	3.6	4.2	18.6	NA	2.2
35	5 × 9	0.0	−100.0	4.0	4.7	18.9	NA	2.9
36	WE1101	0.9	54.8	2.7	2.7	19.4	0.9	2.3
37	DK8031	0.8	32.5	3.2	3.7	18.7	0.6	38.3
38	H516	0.5	−22.2	2.9	3.2	19.6	0.5	20.9
39	Duma43	0.2	−72.4	3.4	4.0	18.5	0.1	21.0

*MLN-early* MLN score 21 days post inoculation, *MLN-late* MLN score 42 days post inoculation, *GY* grain yield, *MOI* percent of moisture at harvest, *EPP* ear per plant, *ER* ears rot

### Phenotypic correlations

**Fig. 3:**
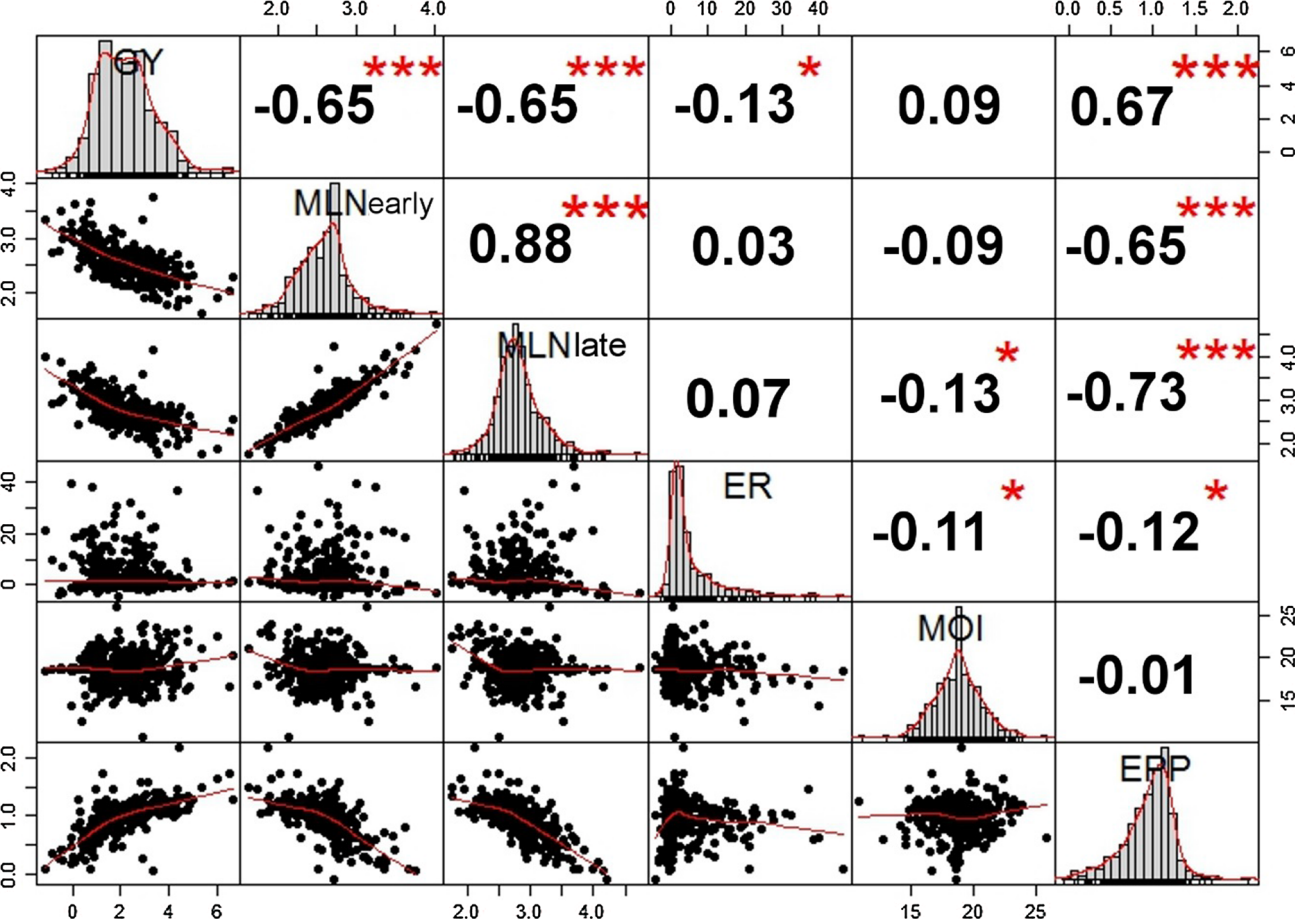
Phenotypic correlations among traits for 344 hybrids evaluated over 2 years at the MLN screening site in Naivasha, Kenya

Coefficients of phenotypic correlation were significant among most traits (Fig. 3), being highest with MLN scores at early and late stages (r = 0.88, *P* < 0.001). MLN scores were negatively correlated with all traits except ear rot that had the highest correlation with number of ears per plant (r = −0.73, *P* < 0.001). Grain yield was highly negatively correlated with MLN scores (r = −0.65, *P* < 0.001) and moderately negatively correlated with ear rot (r = −0.13, *P* < 0.05), but had a significant positive correlation with ears per plant (r = 0.67, *P* < 0.001).

## Discussion

Understanding genetic differences for MLN tolerance, yield, and agronomic traits, as well as the type of gene action involved is useful for prioritizing inbred lines for use as breeding parents or for hybrid formation. The ideal segregating population from such crosses has a large genetic variance combined with a favorable mean, as this enables maximum potential selection gain (Bernardo 2010). A wide range of phenotypic values was observed for most traits, indicating a solid basis for future improvements through breeding. In our study, high genotypic variance and intermediate to high broad-sense heritabilities were observed for MLN severity at early and late observation time points. Relatively high heritability was also observed for grain yield. In contrast to the current study, several studies had reported lower genotypic variances and heritabilities for grain yield under stressed conditions (Atlin and Frey 1990; Bänziger et al. 1997; Beyene et al. 2013).

Genotype × environment interaction for grain yield, disease resistance and agronomic traits in maize has been widely studied. In the current study, both variance of genotype and G × year interactions for all traits were significant suggesting that phenotypic expression of traits for entries evaluated was influenced by the year in which they were grown. However, genotypic variance were 2.5, 2.6 and 3.3 times higher than G × year interactions for MLN score, grain yield, and ear per plant, respectively.

General combining ability and SCA estimates can be useful for choosing breeding parents since they provide information about the potential parental value in crosses as well as describing gene action. GCA mainly relates to additive genetic effects and genes with such effects accumulate as cyclic selection progresses (Hallauer and Miranda 1988). In the current study, GCA estimates for MLN resistance, ear rot and ear per plant were 2.2–3.5 times higher than SCA estimates, indicating that additive gene action is more important than non-additive gene action in regulation of MLN resistance in the germplasm evaluated. Crosses involving parents with high GCA often had favorable SCA for MLN severity and GY. For example, crosses, “CKDHL120918 × CKLTI0136”, “CKLTI0136 × CKLMARSI0029” and “CKDHL120694 × CKDHL120918” had the best SCA effect for grain yield, MLN severity, ears per plant, and ear rot. The above crosses involved best × best or best × poor combining parents. There have been no reports to date describing the mode of gene action regulating MLN resistance, but genetic regulation of individual maize viruses has been reported (Redinbaugh and Pratt 2009). Nelson et al. (2011) observed that the level of resistance to MCMV varies widely among maize lines tested in Hawaii and suggested that MCMV resistance in maize is a quantitative trait.

Results of the current study indicate that significant genetic variation for resistance to MLN exists in tropical maize (Table 3). Five MLN resistant inbred lines (CKDHL120918, CKTI0137, CKTI0138, CML494, and CKDHL500) had negative and significant GCA for MLN early and late severity scores and positive GCA scores for GY (Table 3). Nelson et al. (2011) reported that almost all temperate climate inbred lines and hybrids are highly susceptible to MCMV. However, resistance to various potyviruses has been identified in maize germplasm from North America, the Caribbean, South America, Asia, and Africa (Louie et al. 1990; Brewbaker et al. 1991). Loci for resistance to MDMV, WSMV, and SCMV have been designated as *Mdm1, Wsm1 and Scm1* and have been mapped to the short arm of chromosome 6 (McMullen and Louie 1989).

Selection of parental lines is an important step in the development of superior high yielding cultivars. Methods that could predict single-cross maize hybrid performance with high level accuracy prior to field evaluation are of particular interest in crop improvement programs. Predicting performance of hybrids from per se performance of their parental inbred lines has been unsuccessful due to masking dominance effects (Hallauer 1990). Best linear unbiased prediction (BLUP) was investigated by Bernardo (1996) to predict performance of untested single crosses using phenotypic information of related single crosses and genetic relationships among their parental inbred lines. The GCA estimates of parental lines provide an established and simple approach to predict hybrid performance (Melchinger et al. 1987). In this study, GCA based prediction of 28 × 28 maize inbred lines crosses was performed to predict single-cross performance for MLN resistance and GY. Using GCA estimates of MLN susceptibility to predict hybrid MLN resistance accounted for up to 90% of the variations in our study. The GCA estimates of MLN susceptibility also effectively predicted hybrid grain yield under MLN pressure, accounting for 67% of the variations. These results suggest that GCA-based prediction can be used to predict MLN resistance and grain yield prior to field evaluation, thus significantly reducing the cost of variety development for tolerance to MLN disease.

One of the main objectives of this experiment was to identify high-yielding MLN tolerant single cross hybrids to be used as seed parents in three-way cross hybrids and/or for commercial production after testing in successive multi-location testing. Thirty single crosses with average grain yield greater than 4 t/ha were identified. The top 10 hybrids produced 684–1008% higher GY than the mean of the commercial checks (Table 4). Among the top yielding single cross hybrids, CKDHL120918, CKLTI0227 and CML550 each appeared in seven combinations, followed by CKDHL0500 (five), CML494 (four) CKLTI0139 and CKLTI0138 (three). These parents have negative GCA for MLN tolerance and positive GCA for grain yield under MLN artificial inoculation. The GCA is the most important indicator of potential for inbred lines in hybrid combinations (Poehlman 1979).

It is particularly relevant to report that seven of the inbred lines used in this study involve CML494 as an immediate parent. This is important since all of these lines are derived from BC_1_ and they share a higher coefficient (75%) of parentage than the other lines in the study. They are expected to have lower heterosis in combination with each other than in combination with less related lines. The inbreeding depression of these single cross hybrids somewhat confounds the interpretation of the GCA estimates for GY and MLN susceptibility for CML494 and derived lines. It is expected that GY and MLN susceptibility GCA estimates of these lines would be improved in combination with lines which are less related. At the same time, related line crosses can be very useful as single cross seed parents for three way hybrid formation since they are expected to have higher heterosis with elite lines from the opposing heterotic group. Therefore, it would be expected that some of the CML494 related line single crosses from the current study may have excellent GCA in three way cross combinations with Heterotic Group A testers which needs further investigation. The MLN tolerant inbred lines identified in this study will be useful for improving the levels of MLN tolerance in both public and private sector tropical maize breeding programs. In another study, using lines identified in this study, over 1000 new DH lines derived from the resistant × resistant crosses have been developed. These will be useful to develop new MLN tolerant hybrids.

Pearson’s correlation coefficients between MLN disease severity and grain yield under MLN inoculation was significant and negative *(*−0.65*, P* < 0.001*)* confirming the well-established correlation between disease resistance and grain yield in maize. These correlations indicate that MLN rating can be used as selection criteria in developing high yielding MLN tolerant hybrids. In most cases, GY exhibited higher error variances and/or lower heritability estimates than MLN rating. Furthermore, MLN disease rating can be used for discarding susceptible germplasm at early screening stages before tolerant materials go into expensive multi-location testcross trials. Visual rating of MLN has been effective and efficient for screening large numbers of entries (Semagn et al. 2015; Mahuku et al. 2015). CIMMYT and its partners used visual screening as a preliminary step leading to the eventual release of four MLN tolerant hybrids for commercial production in Kenya. Several new hybrids are in National Performance Trials in Uganda and Tanzania, a step required for commercialization in these countries.

## Conclusion

A diallel experiment of 340 single cross hybrids and four commercial checks were evaluated for 2 years under artificial MLN inoculation. Combining ability estimates indicate a prevalence of additive gene action rather than non-additive gene action and thus rapid progress can be expected from recurrent selection. MLN tolerant inbred lines and singe cross hybrids identified in this study could be used in breeding programs to develop MLN tolerant hybrids in East Africa where MLN has become a serious threat to maize production. In addition, 30 MLN singe cross hybrids that gave greater than 4 t/ha under MLN inoculation were identified. These single cross seed parents will be used for making producible three-way cross hybrids which combine multiple abiotic and biotic stress tolerance traits and are affordable for resource constrained farmers in eastern Africa.

## References

[CR1] Adams IP, Harju V, Hodges T (2014). First report of maize lethal necrosis disease in Rwanda. New Dis Rep.

[CR2] Atlin GN, Frey KJ (1990). Selecting oat lines for yield in low-productivity environments. Crop Sci.

[CR3] Bänziger M, Betrán FJ, Lafitte HR (1997). Efficiency of high-nitrogen selection environments for improving maize for low-nitrogen target environments. Crop Sci.

[CR4] Bernardo R (1996). Best linear unbiased prediction of maize single-cross performance. Crop Sci.

[CR5] Bernardo R (2010). Genomewide selection with minimal crossing in self-pollinated crops. Crop Sci.

[CR6] Beyene Y, Mugo SN, Mutinda C (2011). Genotype by environment interactions and yield stability of stem borer resistant maize hybrids in Kenya. Afr J Biotechnol.

[CR7] Beyene Y, Mugo SN, Semagn K (2013). Genetic distance among doubled haploid maize lines and their testcross performance under drought stress and non-stress conditions. Euphytica.

[CR8] Brault V, Uzest M, Monsion B (2010). Aphids as transport devices for plant viruses. C R Biol.

[CR9] Brewbaker JL, Kim SK, Logrono ML (1991). Resistance of tropical maize inbreds to major virus and virus-like diseases. Maydica.

[CR10] Cabanas D, Watanabe S, Higashi CHV, Bressan A (2013). Dissecting the mode of maize chlorotic mottle virus transmission (Tombusviridae: Machlomovirus) by Frankliniella williamsi (Thysanoptera: Thripidae). J Econ Entomol.

[CR11] Castillo J, Hebert TT (1974). Nueva enfermedad virosa afectando al maiz en el Peru. Fitopatologia.

[CR25] CRP Maize (2013) Leading the fight against maize lethal necrosis. CRP annual report, 2013

[CR12] De Groote H, Oloo F, Tongruksawattana S, Das B (2016). Community-survey based assessment of the geographic distribution and impact of maize lethal necrosis (MLN) disease in Kenya. Crop Prot.

[CR13] Fan XM, Zhang YD, Yao W (2014). Reciprocal diallel crosses impact combining ability, variance estimation, and heterotic group classification. Crop Sci.

[CR14] FAOSTAT (2014) http://Faostat.fao.org

[CR15] Gowda M, Das B, Makumbi D (2015). Genome-wide association and genomic prediction of resistance to maize lethal necrosis disease in tropical maize germplasm. Theor Appl Genet.

[CR16] Griffing B (1956). Concept of general and specific combining ability in relation to diallel crossing systems. Aust J Biol Sci.

[CR17] Hallauer AR (1990). Methods used in developing maize inbreds. Maydica.

[CR18] Hallauer AR, Miranda FJB (1988). Quantitative genetics in maize breeding.

[CR19] Handley JA, Smith GR, Dale JL, Harding RM (1998). Sequence diversity in the coat protein coding region of twelve sugarcane mosaic potyvirus isolates from Australia, USA and South Africa. Arch Virol.

[CR20] Jensen SG (1991) Seed transmission of maize chlorotic mottle virus.PDF

[CR21] Louie R (1980). Sugarcane mosaic virus in Kenya. Plant Dis.

[CR22] Louie R, Knoke JK, Findley WR (1990). elite maize germplasm: reactions to maize dwarf mosaic and maize chlorotic dwarf viruses. Crop Sci.

[CR23] Lukanda M, Owati A, Ogunsanya P (2014). First report of *Maize chlorotic mottle virus* infecting maize in the democratic Republic of the Congo. Plant Dis.

[CR24] Mahuku G, Lockhart BE, Wanjala B (2015). Maize lethal necrosis (MLN), an emerging threat to maize-based food security in sub-Saharan Africa. Phytopathology.

[CR26] McMullen MD, Louie R (1989). The linkage of molecular markers to a gene controlling the symptom response in maize to maize dwarf mosaic virus. Mol Plant Microbe Interact.

[CR27] Melchinger AE, Geiger HH, Seitz G, Schmidt GA (1987). Optimum prediction of three-way crosses from single crosses in forage maize (*Zea mays* L.). Theor Appl Genet.

[CR28] Nault LR (1978). Transmission of maize chlorotic mottle virus by chrysomelid beetles. Phytopathology.

[CR29] Nelson S, Brewbaker J, Hu J (2011). Maize chlorotic mottle. Plant Dis.

[CR30] Niblett CL, Claflin LE (1978). Corn lethal necrosis - a new virus disease of corn in Kansas. Plant Dis Rep.

[CR31] Poehlman JM (1979). Breeding field crops.

[CR32] Redinbaugh MG, Pratt RC (2009). Virus resistance. Handbook of maize: its biology.

[CR33] SAS Institute (2009) SAS^®^ 9.2 for Windows

[CR34] Semagn K, Beyene Y, Babu R (2015). Quantitative trait loci mapping and molecular breeding for developing stress resilient maize for sub-Saharan Africa. Crop Sci.

[CR35] Shiferaw B, Prasanna BM, Hellin J, Bänziger M (2011). Crops that feed the world 6. Past successes and future challenges to the role played by maize in global food security. Food Secur.

[CR36] USDA (2014). Kenya maize lethal necrosis - The growing challenge in Eastern Africa.

[CR37] Uyemoto JK (1983). Biology and control of maize chlorotic mottle virus. Plant Dis.

[CR38] Wangai AW, Redinbaugh MG, Kinyua ZM (2012). First report of *Maize chlorotic mottle virus* and maize lethal necrosis in Kenya. Plant Dis.

[CR39] Xie L, Zhang J, Wang Q (2011). Characterization of maize chlorotic mottle virus associated with maize lethal necrosis disease in China. J Phytopathol.

[CR40] Zhang Y, Zhao W, Li M (2011). Real-time TaqMan RT-PCR for detection of maize chlorotic mottle virus in maize seeds. J Virol Methods.

